# Labour management and Obstetric outcomes among pregnant women admitted in latent phase compared to active phase of labour at Bugando Medical Centre in Tanzania

**DOI:** 10.1186/1471-2393-14-68

**Published:** 2014-02-12

**Authors:** Clotrida Chuma, Albert Kihunrwa, Dismas Matovelo, Marietha Mahendeka

**Affiliations:** 1 Department of Obstetrics & Gynecology, Catholic University of Health & Allied sciences, P.O.BOX 1464, Mwanza, Tanzania; 2 Department of Obstetrics & Gynecology, Bugando Medical Centre, P.O.BOX 1370, Mwanza, Tanzania

**Keywords:** Latent phase of labour, Active phase of labour, Interventions, Low risk pregnancy

## Abstract

**Background:**

Interventions given to women admitted in latent or active phase of labor may influence the outcomes of labor and ameliorate complications which can affect the mother and fetus. Labour management, maternal and fetal outcomes among low risk women presenting both in latent phase and active phase of labour in Tanzania have not recently been explored.

**Methods:**

This was a descriptive cross-sectional study. It was done from February to April 2013. Case notes were collected serially until the sample size was reached. A structured checklist was used to extract data. Data was analyzed using SPSS version 17. A p < 0.05 was considered significant at 95% confidence interval.

**Results:**

Five hundred case notes of low risk pregnant women were collected, half of each presented in latent phase and active phase of labour. Key interventions including augmentation with oxytocin, artificial rupture of membranes and caesarean section were significantly higher in the latent phase group than the active phase group 84(33.6%) versus 52(20.8%) p < 0.05; 96(38.6%) versus 56(22.4%) p < 0.05 and 87(34.8%) versus 60(24.0%) p < 0.05 respectively. Spontaneous vertex delivery was higher among pregnant women admitted initially in active phase than in latent phase groups 180(72.0%), versus 153(61.2%) p > 0.01). There were more women in the active phase group who sustained genital tract tear and postpartum haemorrhage than in the latent phase group 101(18.6%), versus 38(15.6%) p < 0.01 and 46(18.4%), versus 17(6.6%) p < 0.05 respectively.

**Conclusions:**

Pregnant women admitted at BMC in latent phase of labour are subjected to more obstetric interventions than those admitted in the active phase. There is need to produce guidelines on management of women admitted in latent phase of labour at BMC to reduce the risk of unnecessary interventions.

## Background

The first stage of labor encompasses the onset of labor to the complete dilatation of the cervix, and is subdivided into latent and active phases. The latent phase is the time when the cervix starts to efface and dilate up to 3 cm. While the active phase begins when the rate of cervical dilatation accelerates, which occurs at 4 cm to 10 cm [1].

Despite the fact that Bugando Medical Centre (BMC) is using the modified partograph; there are no management guidelines or protocols on how to manage pregnant women admitted in the latent phase of labour. In this study the low risk pregnancy was defined as any pregnant woman who had no medical problems associated with the pregnancy such as diabetes mellitus, epilepsy, anemia, hypertension, premature labor, previous caesarean section, multiple pregnancies, malpresentation and infections which present a potential risk to the baby. A study indicates that latent phase is a sensitive period that can be influenced by pregnancy and may in turn influence both active and the expulsive phase of labor [2]. Patients in labor are usually admitted to the hospital during the first stage of labor. It is important to differentiate between the active and latent phases because women admitted in latent labor tend to spend more time in the labor ward and have more interventions than those who are admitted during the active phase. When a pregnant woman is admitted during the latent phase of labor, physicians should set reasonable expectations for labor progress to avoid unnecessary interventions and anxiety [1]. The widespread use of routine medical interventions in labor is of worldwide concern [3]. Evidence demonstrates that management of early labor has an impact on maternal and neonatal outcomes, in which women who are admitted in the active phase of labor at 4cms or more cervical dilatation experience less interventions and complications than those admitted in the latent phase of labor with 3cms cervical dilatation or less [4]. Delayed-admission in labor may help to avoid premature and unnecessary intervention in women with prolonged latent phase. A study done by McNiven et al. showed that women who delayed admission while in labor had significant less oxytocin use compared with early admitted ones 40% versus 23%, and shorter duration of labor in hospital 13.5 hours versus 8.3 hours respectively but there was no significant differences in caesarean delivery and neonatal outcomes [5]. Possible reason for the increased rate of intervention is that prolonged latent phase may be misdiagnosed as a protraction or arrest disorder. Prolonged latent phase is associated with a higher risk of subsequent labor abnormalities, such as postpartum hemorrhage, chorioamnionitis and neonatal admission to the intensive care unit and long hospital stay [6].

Factors that may affect duration of the latent phase include unfavorable cervical condition, false labor, sedation and analgesia/anesthesia [5]. Women presenting in the latent phase of labor experienced more caesarean deliveries and active phase arrests of labor than women presenting in active labor. It is uncertain whether inherent labor abnormalities result in latent phase presentation and subsequent physician intervention or whether early presentation and subsequent physician intervention are the causes of the labor abnormalities [7].

## Methods

The study was a descriptive cross-sectional study conducted at Bugando Medical Centre (BMC) which is on the northwest side of Tanzania in the city of Mwanza from February to April 2013. The centre serves as a referral consultant hospital and as a University teaching hospital. The average number of deliveries is 600 per month and it caters primarily for high-risk pregnant women referred from peripheral hospitals and low risk pregnant women residing in Mwanza city.

The study population included low risk pregnant women at term (gestation age 37–42 weeks) aging between 18–35 years, singleton pregnancy and cephalic presentation. Women with the following conditions were considered high risk and were excluded, this included women with multiple pregnancies, previous caesarian delivery, any other presentations different from cephalic, gestation age below 37 weeks, abnormal placentation recorded during antenatal care by ultrasonography, antepartum hemorrhage observed antenatally, and chronic medical conditions (hypertension, asthma, diabetes mellitus, epilepsy, anemia, HIV and sickle cell disease). Serial sampling method was used until a desired sample size was reached and the sample size for this study was calculated by using a formula for a difference proportions (equal sized groups).

Latent phase of labour was defined as the interval from when the woman perceives mild regular uterine contractions up to when the cervical dilatation was 3 cm. The active phase of labor was defined as the interval after the latent phase to full cervical dilatation.

Case notes of all eligible women were reviewed and the information extracted using structured checklist. Each case note and checklist was assigned an identification number. Information collected included socio-demographic characteristics and obstetric history such as gravidity, parity and gestational age; interventions such as artificial rupture of membranes, augmentation with oxytocin and caesarean section; maternal and neonatal outcomes including birth weight, APGAR score and admission to Neonatal Care Intensive Unit.

Data was analyzed by using SPSS version 17. Categorical variables were summarized into proportions and percentages. Numerical data was summarized into means, median and standard deviations. Chi-square was used to compare the differences between the two groups for categorical variables while *t*-test was used for continuous variables. Odds ratio was calculated as a measure of the strength of the associations between variables. Ethical review and approval to conduct the study was obtained from the department of Obstetrics and Gynecology, Catholic University of Health & Allied Health sciences (CUHAS) and Bugando Medical Centre (BMC) Research Ethics Committee. The filled checklists were kept in secure place for confidentiality after data entry, cleaning and dissemination of results.

## Results

During the study period 2,059 pregnant women delivered and of these 500(24.3%) met the inclusion criteria (250 in latent phase and 250 were in active phase of labor). Most participants had age ranging between 18–40 years with mean age of 25.42 ± 5.25 years. Majority of women in both active and latent phases of labor were in the age group between 20–35 years 222(88.8%), and 208(83.2%) respectively and majority with only primary school education (179(71.6%), and 199(79.6%)) (Table 1).

**Table 1:** Socio-demographic and Obstetric characteristics of parturients in latent and active phase of labor (N = 500)

**Characteristics**	**Latent phase n (%)**	**Active phase n (%)**
**Age**		
<20	26 (10.4)	34 (13.6)
20-35	222 (88.8)	208 (83.2)
>35	2 (.8)	8 (3.2)
**Level of education**		
No formal education	6 (2.4)	12 (4.8)
Primary education	179 (71.6)	199 (79.6)
Secondary education	65 (26.0)	39 (15.6)
**Gravidity**		
Primigravida	120 (33.9)	234 (66.1)
Multigravida	130 (89.0)	16 (11.0)
**Parity**		
0	122 (48.8)	88 (35.2)
2-4	120 (48.0)	148 (58.8)
≥5	8 (3.2)	15 (6.0)

Among the total of 500 women in the study, 354(71%) were primigravida 120(34%) admitted in latent phase and 234(66%) in active phase of labour (Table 1).

Proportion of pregnant women who received key interventions including augmentation with oxytocin, artificial rupture of membranes and caesarean section were significantly higher in the latent phase group than in the active phase group 84(33.6%) versus 52(20.8%) p < 0.05; 96(38.6%), versus 56(22.4%) p < 0.001 and 87(34.8%), versus 60(24.0%) p < 0.05 respectively. In both groups the most frequent indication for caesarean section was fetal distress however this intervention was done more among the latent phase group 61(24.4%), versus 25(10.0%) p < 0.05 (Table 2).

**Table 2:** Association between phases of labour with interventions, mode of delivery, maternal complications and fetal outcomes (N = 500)

**Characteristics**	**Latent phase**	**Active phase**	**OR**	**CI**	***χ***^**2**^	**p-value**
**Oxytocin use**						
Yes	84 (33.6)	52 (20.8)	1.956	1.715	10.343	0.001
No	166 (66.4)	198 (79.2)				
**Artificial rupture of membranes**						
Yes	96 (38.6)	56 (22.4)	1.794	1.580	15.124	<0.0001
No	154 (61.6)	194 (77.6)				
**C/S**						
Yes	88 (35.2)	60 (24.0)	1.759	1.190	9.333	0.002
No	162 (64.8)	190 (76.0)				
**Reasons for c/s**						
**Fetal distress**						
Yes	61 (24.4)	25 (10.0)	3.421	1.711	12.525	0.001
No	26 (75.6)	35 (90.0)				
**CPD**						
Yes	13 (5.2)	17 (6.8)	0.515	0.233	2.751	0.097
No	72 (94.8)	42 (93.2)				
**Obstructed labour**						
Yes	14 (5.6 )	18 (7.2)	8.999	6.445	8.706	0.013
No	76 (94.4)	41 (92.8)				
**Mode of delivery**						
**SVD**						
Yes	153 (61.2)	179 (72.0)	0.589	0.404	7.607	0.006
No	97 (38.8)	71 (28.0)				
**Assisted vaginal delivery**						
Yes	10 (4.0)	11 (4.4)	1.105	0.461	11.207	0.824
No	240 (96)	239 (95.6)				
**Maternal complications**						
**Genital tears**						
Yes	38 (15.6)	101 (18.6)	8.999	6.445	8.706	0.013
No	212 (84.4)	149 (81.4)				
**PPH**						
Yes	17 (6.6)	46 (18.4)	20.222	11.899	9.945	0.001
No	223 (93.4)	204 (81.6)				
**Apgar scores**						
≤7	48 (19.2)	35 (14.0)	0.891	0.564	6.072	0.108
>7	202 (80.8)	215 (86.0)				
**Neonatal ICU admission**						
Yes	32 (12.8)	23 (9.2)	0.152	1.521	2.033	0.362
No	218 (87.2)	227 (90.8)				

Spontaneous vertex delivery was significantly higher in the active phase group than the latent phase group, 180(72.0%) against 153(61.2%) p < 0.01. There was no difference with regard to proportions of women who had assisted vaginal delivery between the two groups. In terms of maternal complications, there was more genital tract injury and postpartum hemorrhage (PPH) in the active phase than in the latent phase group 101(18.6%), versus 38(15.6%) p < 0.01) and 46(18.4%) versus 17(6.6%) p < 0.05) respectively (Table 2).

There was no significant difference in fetal outcomes with regard to Apgar score and admission to the Neonatal Intensive Care Unit between the two groups (Table 2).

## Discussion

The need to minimize medical or surgical interventions among pregnant women in labour is a challenge in most of clinical settings. This has led to never-ending debate among scholars between natural childbirth and the techno-medical model of childbirth.

In this study, most primigravida women presented in the active phase of labor as opposed to multigravida women who presented in latent phase. This can be attributed to previous experience among multiparous women which prompt them to seek medical care earlier. These findings are not comparable to other similar studies due to differences in population size which was small and obstetrics characteristics which involved primigravida only. Also studies in Bangladesh and Iraq had the cut-off point of 4 cm for both latent and active phase which might have affected the categorization and hence the difference in the outcome from the study [8,9].

The study found that women who were admitted in their latent phase of labor have increased obstetric interventions compared to those in active phase of labor, the findings which are similar to the previous studies done in Scotland and USA [3,7]. The most frequent interventions were augmentation with oxytocin which was high in women admitted in latent phase than active phase of labor (33.6% vs 20.8 p < 0.05). This was similar to other studies done in Iraq (58.3% vs 41.5% p < 0.05) [9] and Columbus (80.4% vs 48.9%, p < 0.05) [9,10]. However, these findings are different from the study done in Iran which found that the rate of oxytocin augmentation was similar in both groups [4]. In this study, the interventions could have been undertaken to avoid adverse maternal and fetal outcomes, whereas a study in Sweden attributed this to patients’ self initiative for intervention to avoid labor pain [2]. Similar to the study in Columbus, the rates of amniotomy was more in the latent phase group than in the active phase groups 51.1% vs 41.3% p < 0.05 [10]. Caesarean section was more in latent phase compared to active phase of labor group in this study and other similar studies done in Iran, USA and Ethiopia [4,7,11]. But reasons for caesarean section were different. The main indication for caesarean section in this study was fetal distress whereas dystocia was the leading indication in a study done in Iraq [9]. This is because the study in Iraq involved only primigravida while this study included both primigravida and multiparous women. Fetal distress in this study was diagnosed by listening to the fetal heart rate using a pinard stethoscope and by examining the state of the liquor. Confirmation of the fetal distress by fetal scalp pH estimation is not a practice at BMC thus a large window of validity for the diagnosis may exist.

In this study there was no difference in the proportion of women who had assisted vaginal delivery. This is similar to a study done in USA [7]. Regarding mode of delivery, studies in Bangladesh and Ethiopia found that normal vaginal delivery was significantly higher in active phase of labor than in latent phase of labor (p < 0.05), the finding which relate to this study (p < 0.006) [8,11].

Similar to the studies in Iraq and USA [7,9], there was no statistical significant difference in fetal outcomes in terms of Apgar score and admission in Neonatal Intensive Care Unit between newborns delivered by women in latent compared to those delivered by women in active phase of labor. Since there was no difference in fetal outcomes between the two groups one may argue that a subset of women in latent phase group received unnecessary interventions.

On assessment of maternal complications, this study showed that more women had perineal tear and PPH in the active phase group than those in the latent phase group, but contrary to this, a study in Bangladesh observed more cases of PPH (5.7%) in latent phase group and none was found in active phase group whereas in USA there were no differences in PPH in the two groups [7,8]. Perineal injury was found in 2(5.7%) and cervical tear was found in 1(2.9%) in latent phase of labor whereas no injury was found in active phase of labor in the study in Bangladesh [8]. PPH in the active phase group in this study occurred largely among primiparous women in approximately 50% of cases; thus the difference in labour complication pattern from other studies could be attributed to human resource constraints. The midwife to patient ratio in labour ward at Bugando Medical Centre is insufficient to allow provision of the standard active management of third stage of labour to every woman who gives birth.

The limitation of this study is worth mentioning. Partograph analysis was not conducted; in which course of labour in relation to the mode of delivery could have been assessed. Thus we do not have information on number of pregnant women who crossed the alert line or action line on the partograph and mean cervical dilatation in both latent phase and active phase of labour.

## Conclusions

Low risk pregnant women admitted at BMC in latent phase of labour are subjected to more obstetric interventions than those admitted in the active phase. However the obstetric outcomes between the two groups are similar. There is a need to produce guidelines on management of women admitted in latent phase of labour at BMC to reduce the risk of unnecessary interventions.

### Ethical approval

Ethical review and approval was sought and obtained from a joint Catholic University of Health & Allied sciences and Bugando Medical Center Research and Publication Committee.

## Consent

All participants were provided with consent information sheet and forms to read and consent for participation, but for non literate women, the consent sheet was read aloud in Kiswahili by the recruiter who was not part of the health care provision team to the woman. After agreeing to participate, her thumbprint was stamped on the consent form to signify her consent.

## Competing interests

The authors declare that they have no competing interests.

## Authors’ contributions

CC: Main author of the study, involved in writing the proposal, data collection and analysis. DM: Involved in design, development of proposal, data collection, analysis and preparation of the manuscript. AK: Involved in developing the proposal, data collection, analysis and preparation of the manuscript. MM: Involved in developing the proposal, data collection and analysis. All authors read and approved the final manuscript.

## Pre-publication history

The pre-publication history for this paper can be accessed here:

http://www.biomedcentral.com/1471-2393/14/68/prepub
